# Concurrent structural and biophysical traits link with immunoglobulin light chains amyloid propensity

**DOI:** 10.1038/s41598-017-16953-7

**Published:** 2017-12-01

**Authors:** Luca Oberti, Paola Rognoni, Alberto Barbiroli, Francesca Lavatelli, Rosaria Russo, Martina Maritan, Giovanni Palladini, Martino Bolognesi, Giampaolo Merlini, Stefano Ricagno

**Affiliations:** 1 Dipartimento di Bioscienze, Università degli Studi di Milano, 20133 Milano, Italy; 2 Amyloidosis Research and Treatment Center, Fondazione IRCCS Policlinico San Matteo, and Department of Molecular Medicine, University of Pavia, 27100 Pavia, Italy; 3 Dipartimento di Scienze per gli Alimenti, la Nutrizione e l’Ambiente, Università degli Studi di Milano, 20133 Milano, Italy; 4 Dipartimento di Fisiopatologia Medico-Chirurgica e dei Trapianti, Università degli Studi di Milano, Milano, Italy; 5 CNR Istituto di Biofisica, c/o Università degli Studi di Milano, 20133 Milano, Italy

## Abstract

Light chain amyloidosis (AL), the most common systemic amyloidosis, is caused by the overproduction and the aggregation of monoclonal immunoglobulin light chains (LC) in target organs. Due to genetic rearrangement and somatic hypermutation, virtually, each AL patient presents a different amyloidogenic LC. Because of such complexity, the fine molecular determinants of LC aggregation propensity and proteotoxicity are, to date, unclear; significantly, their decoding requires investigating large sets of cases. Aiming to achieve generalizable observations, we systematically characterised a pool of thirteen sequence-diverse full length LCs. Eight amyloidogenic LCs were selected as responsible for severe cardiac symptoms in patients; five non-amyloidogenic LCs were isolated from patients affected by multiple myeloma. Our comprehensive approach (consisting of spectroscopic techniques, limited proteolysis, and X-ray crystallography) shows that low fold stability and high protein dynamics correlate with amyloidogenic LCs, while hydrophobicity, structural rearrangements and nature of the LC dimeric association interface (as observed in seven crystal structures here presented) do not appear to play a significant role in defining amyloid propensity. Based on the structural and biophysical data, our results highlight shared properties driving LC amyloid propensity, and these data will be instrumental for the design of synthetic inhibitors of LC aggregation.

## Introduction

Systemic amyloidoses are protein misfolding diseases caused by deposition of proteins as fibrillar aggregates in target organs^1^. In these disorders, the amyloidogenic protein precursor is produced at sites distant from those of deposition, being then transported to the tissues through blood^2^. Light chain amyloidosis (AL) is the most frequent systemic form, with an incidence of approximately 10 cases per million-persons/year; it is caused by deposition of excess monoclonal immunoglobulin light chains (LCs) produced by a bone marrow plasma cell clone^3^. Since the incidence of AL increases with age, the socio-economic impact of this devastating disease is expected to grow within the ageing population of industrialized countries.

AL is a heterogeneous disease, both in terms of causative proteins and of the pattern of organ involvement. The extreme variability among LCs, caused by genetic rearrangement and somatic hypermutation^4^, translates into the fact that virtually every monoclonal LC is unique in its amino acid sequence. The clinical phenotype of AL is also polymorphic, most patients showing multi-organ involvement at presentation^2^: involvement of the heart, in particular, is frequent (~75% of cases) and dictates the prognosis^2,3,5^. A growing body of experimental and clinical evidences from patients with cardiac involvement indicate that damage is not only caused by fibril deposits, but also by pre-fibrillar amyloidogenic LCs, which are themselves directly toxic for target cells^6–9^.

Understanding the specific properties of different LCs in their soluble native state, therefore, appears as a rational approach to explore the determinants of amyloid formation, organ tropism and dysfunction *in vivo*. Because of insufficient insight, no treatments exist yet to block fibril formation and prevent tissues damage. In fact, the current therapeutic strategies are based essentially on halting the production of amyloidogenic LCs from the plasma cell clone by means of chemotherapy^5^.

Structural characterization has shown that LCs assemble into homodimers, each monomer consisting of two immunoglobulin domains^10^, where the N-terminal variable domain (V_L_) displays high sequence variability. In particular, the three hypervariable complementarity determining regions (CDR) that target specific antigens are located in the V_L_ domain. The C-terminal constant domain (C_L_), on the contrary, displays highly conserved sequence within the λ and the κ isotypes. To date, most studies dealing with the biophysical and structural properties of amyloidogenic LCs have focused specifically on V_L_ domains, as they are abundant in fibrils: typically, V_L_ domains belonging to amyloidogenic LCs are thermodynamically and kinetically unstable, while the three-dimensional structures of amyloidogenic and non-amyloidogenic V_L_ studied match closely^11–15^. However, many observations stress the relevance of studying full length LCs: (i) although V_L_ are abundant in fibrils, the full length LCs and C_L_ domains have also been found in deposits^12,16–18^; (ii) so far, FL LCs and not truncated forms, have been found soluble in blood and serum^19^; iii) recent reports underline the relevance of the C_L_ domain in determining both the biophysical properties and the aggregation propensity of LCs^20–23^.

The biophysical characterizations hitherto reported have mostly focused on the pairwise comparisons of one amyloidogenic with one non-amyloidogenic LC^11,16,20,21,24^. At the light of the mentioned sequence variability^4^, the results of such studies may prove difficult to generalize. In an effort to extend our views, here we present a comprehensive biophysical and structural characterization of a pool of eight amyloidogenic LCs originating from different patients (thus endowed with different amino acid sequences). Among all patients who overexpress one LC variant, only a subset develops AL (specific germline genes typically of the λ isotype are overrepresented among amyloidogenic LCs^25–27^), therefore five LCs not displaying amyloid propensity in patients were included as controls in our test pool. We explored the correlation between the aggregation propensity observed in patients and the LC molecular properties that may elicit misfolding, and are held to be associated with proteotoxicity. Our results provide insight on the fundamental molecular properties of pathogenic LCs, and in parallel suggest concepts for the design of therapeutic approaches to AL directly targeting circulating LC molecules.

## Results

### Set-up of the LC test pool

**Figure 1:**
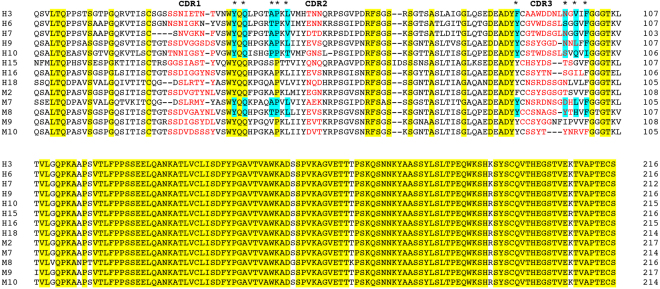
Multialignment of all thirteen LCs used in this work: the residues conserved in all sequences are highlighted in yellow, residues involved in the V_L_ – V_L_ interface in all the crystal structures are highlighted in cyan, while the residues belonging to the three CDRs are shown in red.

In order to explore a most general context, while coping with the issue of high LC sequence variability, we devised an experimental approach based on three criteria. (i) Our biophysical and structural characterization covered thirteen LCs from distinct patients. LCs were distributed in two groups: a set of eight amyloidogenic LCs responsible for severe cardiac involvement and symptoms in patients (hereafter, H LC), and a set of five non amyloidogenic LCs isolated from patients affected by multiple myeloma for which no amyloid aggregation or proteotoxicity was observed in patients (hereafter, M LC) (Table 1 and Table S1). (ii) All the selected LCs were of the λ isotype, which is overrepresented among amyloidogenic LCs (approximately 75%)^2,28^, all the H LCs (except H10 from germline 1–36) belong to germlines commonly found among AL patients^29^. However, the LCs were distributed across different families and germlines to avoid focusing on family/germline-specific properties (Table 1 and Fig. 1). (iii) All proteins used in the experiments were full length LCs, since the blood concentration of these species in each patient directly correlates with the severity of organ dysfunction, particularly regarding the heart (Table S1)^5,8^. Our experiments focused on the properties of native LCs, since ultimately these represent the circulating reservoir of aggregation prone material; LCs were either expressed recombinantly^30^ or purified from patients’ urines (Table 2).

**Table 1:** Biochemical and clinical features of H and M LCs (for more extended information, with a complete set of clinical data, see Table S1).

LC code	Germline	Gender, age	Diagnosis	Organs involved	Serum λ FLC (mg/l)
**H3**	1c (IGLV1-44)	M, 65	AL	H	252
**H6**	1b (IGLV1-51)	F, 72	AL	H, K, PNS	248
**H7**	1b (IGLV1-51)	M, 45	AL	H	477
**H9**	2c GLV2-8)	M, 59	AL	H, ST	699
**H10**	1a (IGLV1-36)	M, 73	AL	H, L	475
**H15**	6a (IGLV6-57)	M, 53	AL	H, PNS, ST	839
**H16**	2a2 (IGLV2-14)	M, 72	AL	H, K	383
**H18**	3 l (IGLV3-19)	M, 69	AL	H, ST, PNS	509
**M2**	2b2 (IGLV2-23)	M, 65	MM	—	1140
**M7**	3 l (IGLV3-19)	F, 71	MM	—	6130
**M8**	2b2 (IGLV2-23)	M, 48	MM	—	573
**M9**	2b2 (IGLV2-23)	M, 61	MM	—	8510
**M10**	2a2 (IGLV2-14)	M, 55	MM	—	12200

Abbreviations: M, male; F, female; MM, multiple myeloma; H, Heart; K, Kidney; ST, Soft Tissues; PNS, Peripheral Nervous System; BJ, Bence Jones protein (monoclonal urinary free light chains); FLC, Free Light Chains.

**Table 2:** Biophysical properties assessed for H and M LCs.

LC	Source	Tm_app_ Far UV (°C)	Tm_app_ Near UV (°C)^1^	ONSET ANS SIGNAL (°C)	Hydrophobicity (%)	Proteolysis^2^	Trypsin sites
**H3**	Rec	(40.2) – 54.4 ± 0.8	(44.5)—53.1	54.8	23.1	+++	14
**H6**	Rec	(43.9) – 54.5 ± 0.8	(45.3)—	55.3	23.1	+++	18
**H7**	Rec	(42.8) – 54.7 ± 0.5	(48.7) – 56.2	54.8	23.6	++	16
**H9**	BJP	52.0 ± 0.8	54.0	55.8	21.3	+++	14
**H10**	BJP	54.9 ± 0.5	54.3	58.3	23.7	++	14
**H15**	BJP	55.6 ± 0.6	—	54.7	21.8	+ +	14
**H16**	BJP	51.3 ± 0.7	51.9	54.5	22.3	+++	13
**H18**	BJP	52.4 ± 0.5	51.1	51.5	22.9	++	13
**M2**	BJP	61.5 ± 0.8	—	63.9	23.0	+	15
**M7**	BJP	54.7 ± 0.5	55.3	58.8	22.4	+	15
**M8**	BJP	72.6	—	73.5	22.7	+	15
**M9**	Rec/BJP	(63.2) – 68.2 ± 1.4	(58.8) – 67.7	62.8	24.2	+	15
**M10**	BJP	58.0 ± 0.6	—	56.1	21.5	+	15

Rec, recombinantly expressed LC; BJP, Bence Jones proteins purified from urines. (^1^): some Tm_app_ values could not be measured due to protein aggregation during the temperature ramp. (^2^): +++:  < 20%, ++: 20–60%, +:  > 60% fraction of uncleaved protein after 180 min.

### LC fold stability

**Figure 2:**
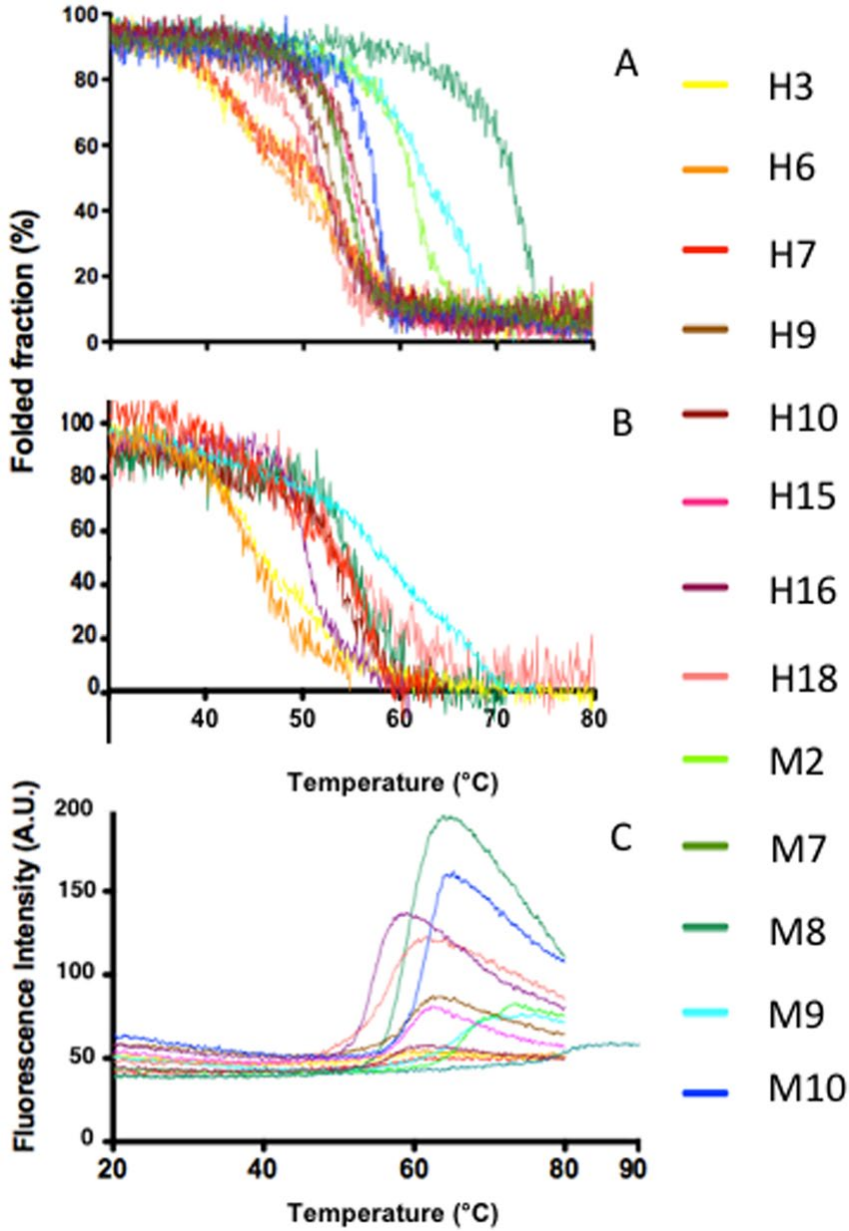
Thermal stability of H and M LCs. Far-UV (**A**) and Near-UV (**B**) CD temperature ramps of the two sets of LCs used in this study. (**C**) Temperature ramps followed using ANS fluorescence. The H and M LCs are shown with warm and cool colours, respectively.

Protein stability within the two LC groups was assessed using three different and complementary approaches. Unfolding temperatures for each LC were monitored by Far-UV circular dichroism (CD), by Near-UV CD, and by 8-anilino-1-naphthalenesulfonic acid (ANS) binding and fluorescence signal (Fig. 2). LC unfolding monitored through these spectroscopic methods provides information on the loss of secondary structure, of tertiary structure, and on the exposure of buried hydrophobic residues to the solvent, respectively. As previously reported the unfolding of full length LCs is not reversible (data not shown)^22^, thus all temperatures corresponding to the unfolding inflection points should be considered apparent melting temperatures (Tm_app_). The Far and Near-UV spectra of all LCs under native conditions are shown in Fig. S1.

In general, H LCs (warm colours in Fig. 2) displayed lower Tm_app_ compared to M LC (cool colours in Fig. 2), as determined from the Far-UV and Near-UV CD temperature ramps shown in Fig. 2A and B, respectively. However, low Tm_app_ values do not correlate perfectly with LCs amyloidogenicity, since M7 and M10 LCs unfold at temperatures close to those of the most stable H LCs (Table 2). Interestingly, most LCs show a one-step unfolding process, but three H LCs (H3, H6, H7) and one M LC (M9) display a two-step process, observed both in Far- and Near-UV temperature ramps (Fig. 2A,B). At the protein concentration required for Near-UV experiments four LCs (H10, M2, M8, M10) precipitated before the unfolding process was complete, thus Tm_app_ could not be determined. Nevertheless, the Tm_app_ values obtained by monitoring the unfolding both by Near-UV and Far-UV CD, show a good agreement (Table 2), indicating that for these LCs the loss of secondary and tertiary structures are simultaneous processes. Student’s test calculated using Tm_app_ values determined by Far-UV indicate that the differences between H and M-LCs are significantly different (P < 0.005).

While the Near-UV CD signal is strongly dependent on Trp residues, and may therefore provide rather local information about the unfolding process, an increase in ANS fluorescence indicates the exposure of hydrophobic residues from the protein core. Inspection of Fig. 2C shows that unfolding monitored by ANS fluorescence is compatible with exposure of the hydrophobic core residues in a one-step process in all the analysed LCs. This observation rules out that stepwise unfolding of the two distinct domains occur in the LCs (H3, H6, H7, M9), which display two-step unfolding when monitored by CD signal; thus the temperatures corresponding to the first inflection points are written in parenthesis in Table 2. The onset of the ANS signal, but not the inflection point of the curves, correlates well with the Tm_app_ values calculated from the CD signals; such apparent discrepancy suggests that the hydrophobic core is exposed only at an advanced state of unfolding, which leads to an (apparent) systematic Tm_app_ overestimation in ANS temperature unfolding. Taken together these data suggest that the loss of tertiary and of secondary structures are simultaneous processes, indicating that LC dimers unfold through a cooperative process. Importantly, the ANS spectra recorded under native conditions yield undistinguishable low signals (data not shown), suggesting that under such conditions none of the LCs studied in this work expose relevant hydrophobic patches to the surface.

### LC dynamics

In order to obtain an indirect assessment of protein dynamics, limited proteolysis of the different LCs using trypsin was performed. Typically, fast proteolysis kinetics correlate with marked protein dynamics; conversely, protein rigidity correlates with slow proteolysis kinetics. In our hands, many but not all of the LCs belonging to the explored set displayed an unexpected resistance to proteolysis by trypsin and chymotrypsin (data not shown); controlled shaking (*i*.*e*. simulating shear forces) was tested to increase the kinetics of proteolysis without any effect (data not shown). Thus, in order to perform proteolysis in an adequate time frame, a sub-denaturant concentration of urea (1 M) was added to the protein solutions: under such conditions all LCs displayed CD spectra indistinguishable from those recorded in the absence of 1 M urea (data not shown).

**Figure 3:**
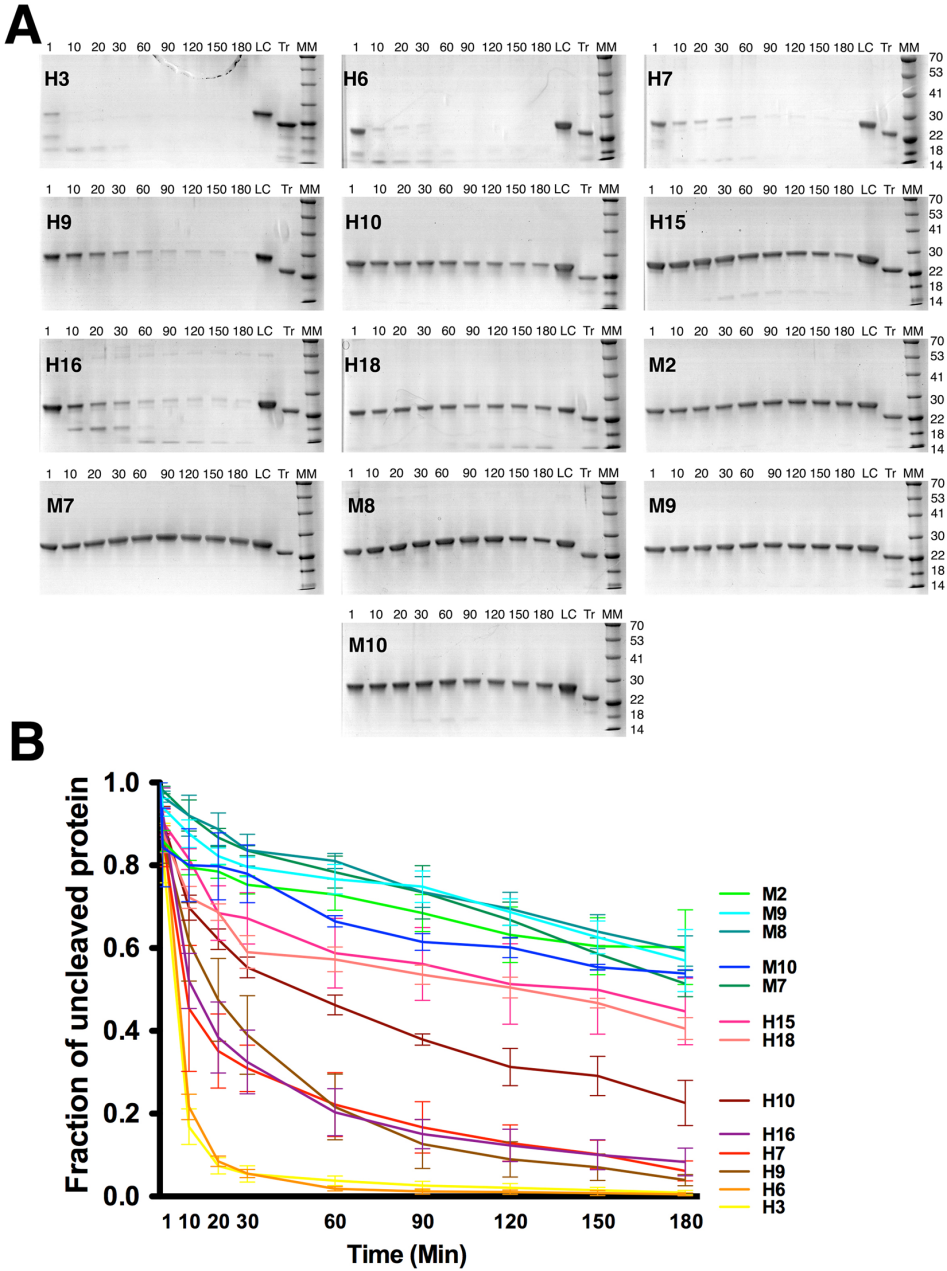
(**A**) SDS-PAGE monitoring the limited proteolysis of H and M LCs by trypsin. The first sample was taken one minute after trypsin addition (1) and then at 10, 20, 30, 60, 90, 120, 150 and 180 min of reaction time. In LC a standard amount of the corresponding LC loaded onto the gel without adding trypsin (Tr). MM indicates molecular markers their mass is expressed in kDa. All SDS-PAGE were run under reducing conditions. Raw images of the all SDS-PAGE are shown in Figure S4 (**B**) Kinetics of LC proteolysis. The intensity of the band corresponding to the uncleaved LC has been quantified at different time points and plotted. As starting point the amount of protein present in the LC sample was chosen. Each curve results from three independent proteolysis experiments. The curves are colour coded as in Figs 2 and 3.

Figure 3 shows the SDS-PAGE monitoring the controlled proteolysis experiments and the plotted fraction of uncleaved protein at different time points. Analysis of the data shows that the kinetics of proteolysis correlate well with amyloid propensity (Fig. 3B): H3, H6, H7, H9, and H16 are almost or totally proteolysed after 60 minutes, and more than 50% of H10, H15 and H18 LCs is cleaved after three hours. This is in stark contrast with the behaviour observed for the five M LCs, which are consistently more resistant to trypsin, in all cases more than 60% of the M LCs remaining uncleaved at the end of the experiment (total of 3 hours). The number of potential trypsin cleavage sites for each LC is reported in Table 2; such numbers vary and do not correlate with the kinetics of proteolysis, suggesting that the observed kinetics underlie genuine differences in protein dynamics or conformational flexibility. Student’s test calculated using the percentage of uncleaved LC at the end of the experiment indicates that the kinetics of proteolysis between H and M-LCs are significantly different (P < 0.0005).

The pattern of proteolysis varied among different LCs. SDS-PAGE gel analysis showed that for most LCs no bands corresponding to specific LC fragments were present, indicating that the LCs had been directly proteolysed to small peptides; although in a few cases discrete bands were detected, they proved unstable over the entire proteolysis experiment (Fig. 3A). Overall, these observations suggest that the proteolytic path is distinct in different LCs, and that no stable LC domains (*e*.*g*. V_L_, C_L_ domains or other fragments) can be isolated during proteolysis under the conditions tested.

### Structural analyses of H and M LCs

**Figure 4:**
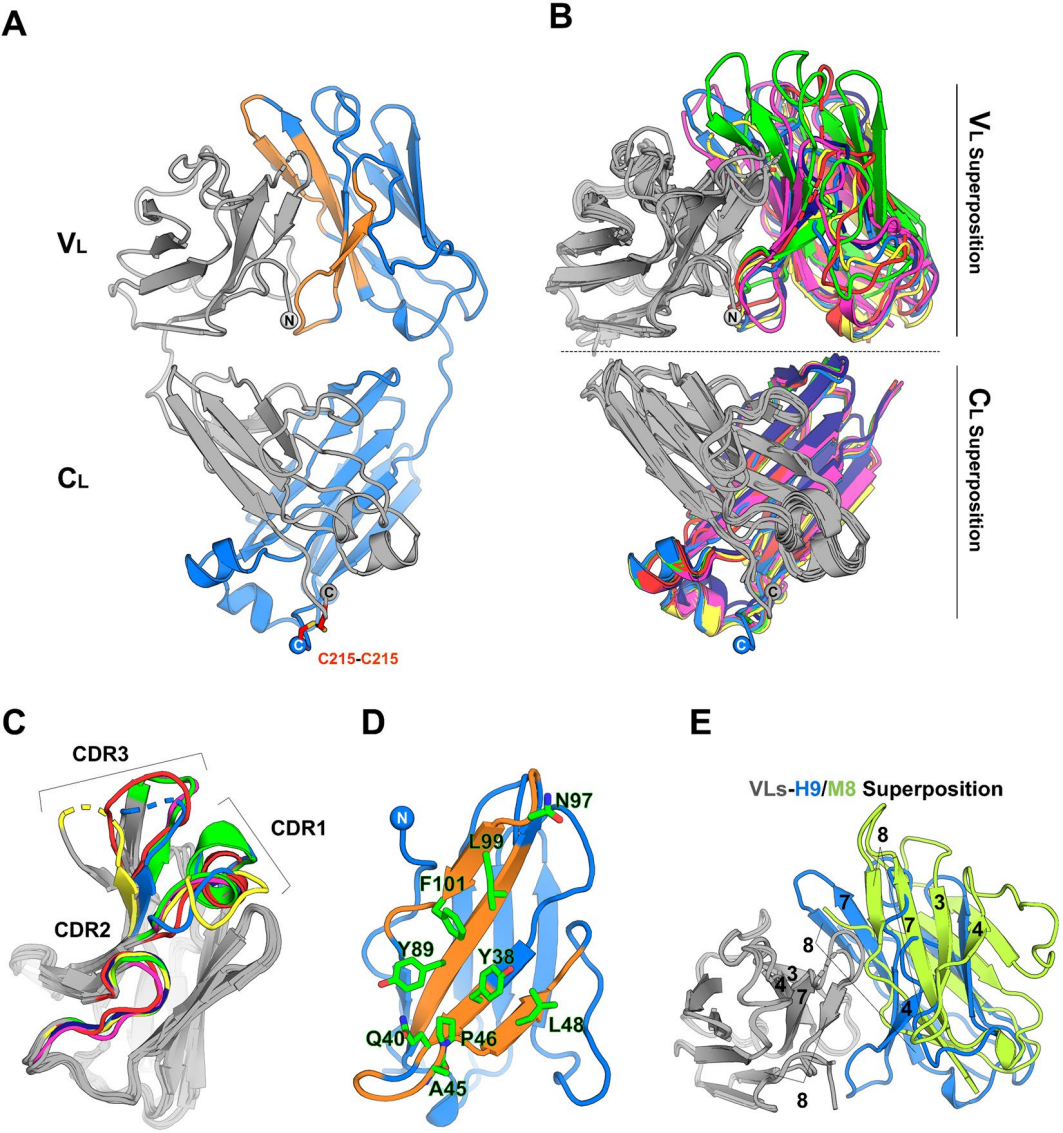
(**A**) Cartoon model of the crystal structure of the H9 homodimer, as representative of the tertiary and quaternary organization of all the LC structures determined in this work. The two LC monomers are coloured in grey and blue. The V_L_ interface region on the blue monomer is coloured in orange. The spheres indicate the position of N/C *termini*. (**B**) Superposition of the dimeric V_L_ (top panel) and C_L_ (bottom panel) domains. One V_L_/C_L_ domain (coloured in grey) was fixed for all the seven structures and the second is coloured according to the different LCs (H3 yellow; H6 green; H7 dark blue; H9 blue; H10 magenta; M7 red; M8 lime green). V_L_ domain from M8 is shown only in panel E. (**C**) Superposition of a single V_L_ domain (grey) from each of the seven LC structures. The complementarity-determining regions (CDRs) belonging to different LCs are coloured following the B panel colour code. (**D**) Cartoon model of a single V_L_ domain from the structure of H9 showing in orange the regions involved in the V_L_ – V_L_ interaction. The residues represented as sticks indicate the positions involved in the V_L_ – V_L_ interface that are conserved among of all LCs structures. (**E**) Cartoon representation of H9 (grey-blue) and of M8 (grey-lime green) where the grey V_L_ are superposed and oriented as in B (top panel). The different orientation of the second V_L_ domain is apparent in the H9 dimer, chosen as an example, compared to the M8 dimer. Labels indicate the β-strand identification number.

In order to gain high-resolution structural insight, all the thirteen LCs were screened for crystal growth. Five H LCs (H3, H6, H7, H9 and H10) and two M LCs (M7 and M8) were successfully crystallized and their 3D-structures solved. Each of the seven LCs crystallized in a different space group, with distinct crystal packing; the crystallographic resolutions achieved ranged from 2.70 Å (H7) to 1.65 Å (H9) (Table S2). The crystal structures were all refined to satisfactory refinement parameters (Table S2); in all cases the LCs were dimeric (Fig. 4A), with an overall quaternary arrangement closely matching that of the previously reported LC dimers^10^. Mass spectrometry and non-reducing SDS-PAGE indicated that 100% of the LC dimers were covalently linked by a disulphide bond located at the chain C-*terminus* (data not shown). However, due to underlying local flexibility, only for H9 a well-defined electron density for such C-terminal intermolecular disulphide bond is visible (Fig. S2).

V_L_ and C_L_ domains display very different structural properties in the seven crystal structures. C_L_ domains (res 115–214) are typically characterized by high quality electron density, are all very well superposable, and the dimeric C_L_–C_L_ arrangement is clearly conserved in all the analysed structures (root mean square deviations -r.m.s.d.- generally fall below 1.0 Å over the entire C_L_–C_L_ dimer - Table S3A and Fig. 4B-bottom). The linker regions connecting V_L_ and C_L_ domains (residues 110–115) allow evident variability in the mutual orientation of V_L_ and C_L_ domains, which is described by the elbow angles (Table S2) spanning from 107.99° (H7) to 157.36° (M8). (Interestingly, the greatest elbow angles were found in the M LCs structures, as for previously reported works^10^. For this reason, the r.m.s.d. values calculated for the full length LCs are not meaningful and are not reported.

Contrary to what has been observed for the C_L_ domains, the electron density quality for the V_L_ domains (residues 1–109) greatly differs among the seven crystal structures. With the only exception of one of the two V_L_ domains of the M7 LC dimer, the two β-sheets building the V_L_ domains are in general easily traceable; however the quality of the electron density for the CDR loops is markedly dependent on the LC crystal structure considered. In H6 and M8 dimers all six CDRs (three for each LC monomer) are traceable in the electron density; in H9 and H10 five out of six are traceable, in H7 three, and in H3 and M7 only two CRDs are traceable. Such different behaviours, which are related to the intrinsic CDR conformational adaptability, do not correlate with LC aggregation propensity or with the flexibility assessed by limited proteolysis. Thus, the observed variety of CDR conformations are likely due to different CDR amino acid sequences, but may also reflect different chemical conditions for crystal growth and different crystal packing.

The above concept is further stressed by the fact the V_L_ domain tertiary structures match closely (Table S3B and Fig. 4C), while conformations of their CDRs are rarely superposable. The inspection of the V_L_ dimer provides a relevant example: the fine details of the V_L_–V_L_ association interface are LC-specific, depending on: (i) sequence variability, which likely causes the mutual reorientation of V_L_ domains by some degrees, as observed in the different structures (Fig. 4B-top); and, (ii) CDR conformational variability (and traceability in the electron density). In some cases (H6, H9, H10) CDR3 (residues 89–97) participates in the interface; both CDR3s in the H7 dimer lack electron density, while in M7 and M8 CDR3 residues are located far from the V_L_–V_L_ interface.

Despite these fine differences, all the structures here presented, except for M8, display an overall conserved V_L_–V_L_ quaternary assembly and association interface (Table S3C and Fig. 4B and E). In the structures of all H LCs and in M7, the association interface is roughly symmetric, and is located on the five-stranded beta sheet (strands 1, 3, 4, 7, 8), where the 3, 4, 7, 8 strands establish the intermolecular contacts. Figure 4D shows the region involved in the V_L_–V_L_ interface together with the most relevant and conserved residues. Only the V_L_–V_L_ interface in M8 is markedly different, one V_L_ domain being rotated and resulting in an asymmetric V_L_–V_L_ interface (Fig. 4E). As for the other structures, in one V_L_ domain of M8 strands 3, 4, 7 and 8 participate to the dimeric interface, while only strands 1 and 8 of the facing/rotated V_L_ domain provide association contacts.

The calculated free energy gain associated with dimer formation, and the resulting dimer interface areas (Table S4), do not correlate with amyloidogenicity. In particular, M8 LC, which is the most stable LC against temperature (Table 2), displays the smallest dimer interface area with the lowest calculated free energy change for quaternary assembly, compared to all other considered LCs (Table S4).

## Discussion

The five M LCs presented in this study are found in patients at extremely high concentrations, much higher than the average concentration of the H LCs (Table 1 and S1), and yet, through the years, the M proteins remain soluble and do not aggregate. Our study aimed to explore the biophysical and structural properties that correlate with LC amyloid aggregation; to reach most general conclusions, we analysed a set of thirteen patient-derived LCs, focusing on some of the commonly proposed structural and biophysical determinants of protein aggregation and toxicity^31^, such as fold stability, protein overall hydrophobicity, protein dynamics and flexibility, and loss or heterogeneity of 3D-structures. The first general conclusion we can draw from the data here presented is that none of such biophysical properties, taken alone, allows clustering of the H *versus* the M LCs; on the contrary, amyloidogenicity seems to stem from the co-existence of more than one of the biophysical factors explored.

According to our assessments of fold stability through CD and ANS fluorescence, the examined H LCs display Tm_app_ values lower than the M LCs; on average the Tm_app_ for H LCs is 53.6 °C *vs*. an average Tm_app_ value of 62.8 °C for the M LCs. Such a trend is mirrored by the average onset temperature for the ANS signal, which is 55.0 °C for H LCs vs. 63.0 °C for the M LCs. Such an overall observation would be in keeping with the idea that amyloidogenicity correlates with lower fold stability, as previously suggested^32^; however, M7 and M10 are notable exceptions showing Tm_app_ values comparable with some of the H LCs. However, M7 was found present at high concentration *in vivo*, and yet no aggregation or toxicity was observed at presentation and during the later follow-up times (Table 1 and Table S1). This marked variability stresses the importance of using large set of proteins and it likely explains the contained differences in Tm_app_ recently observed by others between amyloidogenic and M LCs^23^: Andrich *et al*. reports an interesting biophysical characterisation of nine full length LCs but only four are of the λ isotypes and thus could be compared^23^.

Intriguingly, the low ANS fluorescence observed under native conditions suggests that bulk surface hydrophobicity does not play a main role in determining the H LC toxicity in their native state. Indeed, LC overall hydrophobicity (computed from the amino acid sequences - Table 2) indicates a comparable amount of hydrophobic residues in all thirteen LCs. The Far-UV CD spectra that on average display a slightly more intense signal for M LCs (Figure S1) suggest that typically the latter contain very regular β structure, while H LCs spectra vary more markedly; however, the inference value of these observations is essentially of qualitative nature.

Contrary to the limited information offered by the spectroscopic analyses, controlled proteolysis provided a robust indication that M LCs are more rigid, or less dynamic, than H LCs. These are however divided into two subsets: H3, H6 H7, H9 and H16 are very efficiently proteolysed, while H10, H15 and H18 show slower cleavage kinetics. Inspection of the SDS-PAGE gels shows that the proteolysis pattern is different in different H LCs, suggesting that the sequence of proteolytic events, and not only their kinetics, may be characteristic of each H LC. It is also remarkable that fold stability and kinetics of proteolysis do not necessarily correlate: M7 displays the lowest Tm_app_ among the M LCs, but also very slow proteolysis kinetics; H3, H6 and H9 show relatively high Tm_app_, within the H group, but are almost instantly proteolysed by trypsin. Recently, Morgan *et al*. reported faster kinetics of proteolysis for three amyloidogenic LCs due to kinetic instability of the native state^22^. In the present study we cannot distinguish whether the different kinetics of proteolysis are due to thermodynamic or kinetic instability, which are anyway linked to increased protein dynamics.

Among the experiments here reported, limited proteolysis provided the best correlation between LC behaviour and amyloidogenicity, showing a pronounced proteolytic trend in the H LC group; such consideration may be relevant under different aspects. Increased protein dynamics is considered a risk factor for aggregation propensity^33^. Furthermore, previous studies suggest that the LC fragments released by proteolytic cleavage of full length LCs are more amyloidogenic than the LCs themselves, and may be important species favouring the process of LC aggregation^21,22,34^. Indeed, the existence of LC fragments in amyloid deposits *in vivo* has been uncontrovertibly demonstrated^4,34^. Although the site (*i*.*e*. whether it occurs in the circulation, in the extracellular space or inside cells) and timing (before or after amyloid formation) of LC proteolysis is still a matter of debate, the strong correlation between amyloidogenicity and the ability to be easily proteolysed is stimulating, and suggests that H LCs may release pathogenic peptides much more efficiently than M LCs.

Although crystals could not be grown for the whole set of LCs, the relevant number of crystal structures solved, allows us to extend the discussion to the relationships between the LC biophysical and structural properties. Firstly, and somewhat unexpectedly, the most temperature- and trypsin-sensitive LCs (H3, H6, H7, H9) could be successfully crystallized. Such results indicate that, although characterized by low stability and high dynamics within the group tested, these LCs display a properly folded native structure devoid of large disordered regions that would hamper crystal growth. Secondly, the high level of conservation of the tertiary and quaternary assemblies in the seven LC structures here reported strongly suggests that the differences observed in the biophysical traits mainly depend on sequence variability, and not on major structural rearrangements. Such a consideration would imply that the intrinsic sequence variability, mainly located in the V_L_ domains, does not translate into extended conformational changes but is responsible for the LC behaviour at the molecular level (reflected by the biophysical traits and amyloidogenicity).

Intriguingly, plotting percentage of uncleaved LC at the end of the proteolysis experiments against Tm_app_ (determined by Far-UV) shows that H and M LCs could be clearly clustered (Figure S3). These observations suggest basic criteria for the design of ligands that may decrease LC amyloidogenicity. Molecules acting on the LC dimers, stabilizing the quaternary structure upon binding, as is the case of Tafamidis for the transthyretin tetramer (*i*.*e*. stabilization of the quaternary structure upon binding)^35^, would result in an increased LC fold stability, and in a reduction of overall protein flexibility, the two biophysical traits that were shown here to correlate more strongly with LC amyloid propensity. Specifically, within LC dimers, targeting the dimer region encompassing the V_L_ domains appears as the proper strategy to combat LC amyloidogenicity at its biophysical roots: indeed, small-molecule ligands stabilizing V_L_ dimers have recently been shown to inhibit amyloid formation^36^.

In conclusion, analysis and comparison of the different biophysical properties of H *vs*. M LCs suggests that no single molecular determinant by itself can account for the observed toxicity and aggregation trends, thus stressing the value of biochemical and biophysical studies based on a large pool of proteins rather than on pairwise comparisons. However, fold stability and protein dynamics (as assessed by proteolysis), but not surface hydrophobicity in the native state, or overall 3D-structure rearrangements of the native state, appear to play main roles in determining LC amyloidogenic behaviour. Our study therefore suggests that *in vivo* amyloidogenicity would be the result of concurrent biophysical traits that, as in our pool of proteins, may not necessarily all be present in each toxic LC at the same level.

## Materials and Methods

### Patients’ samples

Urine and bone marrow plasma cells were obtained from patients during routine diagnostic procedures at the Amyloid Research and Treatment Center, Foundation IRCCS Policlinico San Matteo (Pavia, Italy). Acquisition, storage and use of biological samples for research purposes were approved by the Institutional Review Board of Fondazione IRCCS Policlinico San Matteo Pavia; all methods were performed in accordance with the relevant guidelines and regulations. Written informed consent was received from participants prior to inclusion in the study. The presence of tissue amyloid deposits and amyloid organ involvement were defined according to the International Consensus Panel Criteria^37,38^. LC cardiotoxicity was evaluated on the basis of clinical, echocardiography and biochemical parameters^39^ (Table S1).

In parallel to amyloidogenic cardiotoxic LC (H), non-amyloidogenic LC from multiple myeloma patients (M) were used. All the monoclonal LCs included in the study, belong to the λ isotype.

### Cloning of complete monoclonal free LC nucleotide sequences

Total RNA was extracted from 10^7^ bone marrow mononuclear cells using TRIzol reagent (Life Technologies, Paisley, United Kingdom). Monoclonal variable (V_L_) region nucleotide sequences were cloned by an inverse-PCR strategy that preserves the original sequence at 5′ and 3′ ends^40^. The PCR fragment was ligated into the pCR®2.1Vector (TA Cloning Kit; Life Technologies) and cloned into the TOP10 *E*. *coli* cells. After recombinant plasmid purification, insert was sequenced. In order to obtain the original full-length monoclonal LC (variable and constant regions, approximately 650 bp, from codons +1 to +215), standard RT-PCR was employed using the same marrow RNA, a forward patient-specific primer (dictated by codons +1 to +7 of the monoclonal V sequence) and a universal reverse Cλ carboxyterminal cloning primer, corresponding to the last amino acids of the constant region (codons +208 to +215, 5′-TGAACATTCTGTAGGGGCCACTGT-3′). To determine the presumed germline genes of V_L_ regions, sequence alignment was made with the current releases of EMBL-GenBank, V-BASE (V BASE Sequence Directory, MRC Centre for Protein Engineering, Cambridge, UK) and IMGT sequence directories. The gene sequences of the LCs here discussed have been deposited in the GenBank database: KC433670 (H3), KY471433 (H6), KC433671 (H7), KY471435 (H9), KY471432 (H10), KY471436 (H15), KY471437 (H16), KY471434 (H18), KY471441 (M2), KY471438 (M7), KY471439 (M9), KY471440 (M10).

### LC purification from urines

LC were purified to homogeneity from 24 h urine collection. Urines, immediately combined with 0.1% sodium azide (w/v), were centrifuged at 3000 x g for 30 min. Ammonium sulfate was added to the supernatant (65% saturation) and, after overnight incubation, samples were centrifuged at 3000 x g for 30 min. The precipitates were solubilized in 20 mmol/L sodium phosphate, pH 7.0, and dialyzed against the same buffer. All steps were performed at 4 °C. LC were purified by anion exchange chromatography on an AKTÄ Purifier® FPLC system (GE-Healthcare, Piscataway, NJ, USA), using a HiPrep16/10 Q FF column, equilibrated in 20 mM sodium phosphate, pH 7.0. Bound proteins were eluted with a 0 up to 1 M sodium chloride linear gradient. H6, M2, M9 were purified using a cation exchanger column (HiPrep16/10 SP FF), equilibrated in 20 mM Tris-HCl, pH 8.0, and was eluted with a 0 up to 1 M sodium chloride linear gradient. The homogeneity of the isolated species was assessed by 12% SDS-PAGE. The final protein concentration was determined using the Pierce BCA Protein Assay Kit (Thermo Scientific, Rockford, IL, USA) and bovine serum albumin as standard.

### Production of recombinant patient-derived LC

Recombinant LCs of selected patients were produced according to^30^. Briefly, heterologous proteins, produced in the cytoplasm as inclusion bodies, were retrieved and subjected to a renaturation procedure, followed by purification by means of ion exchange and size exclusion chromatography. Recombinant LCs were biochemically characterized by linear MALDI-TOF mass spectrometry and circular dichroism analyses, in order to verify sequence, homogeneity and correct folding. Gel filtration analysis indicates that all LCs used in this work were dimeric in solution (data not shown).

### Circular dichroism spectroscopy

Circular dichroism experiments, in the Far- and Near –UV regions, were carried out on a J-810 spectropolarimeter (JASCO Corp., Tokyo, Japan) equipped with a Peltier system for temperature control. All experiments were carried out in 50 mM sodium phosphate pH 7.4. For the Far-UV region, protein concentration was 0.2 mg/mL in a cuvette with a pathlength of 0.1 cm. Spectra were recorded from 260 to 190 nm, whereas temperature ramps from 20 to 80 °C (monitored wavelength 202 nm, temperature slope 60 °C/hour). Spectra and temperature ramps were performed in triplicate for each LC except for M8 for which no BJ purified material is available. For the Near-UV region, protein concentration was 1 mg/mL in cuvettes with a pathlength 1 cm. Spectra were recorded from 350 to 250 nm, whereas temperature ramps from 20 to 80 °C (monitored wavelength 288 nm, temperature slope 60 °C/hour). Tm_app_ was calculated as the first-derivative minimum of the temperature ramps. Spectra recorded on cooled samples after temperature ramps confirmed that LC dimers unfold irreversibly as previously reported^22^.

### ANS fluorescence

8-Anilino-1-naphthalenesulfonic acid (ANS) binding experiments were carried out at 20 °C and at 0.1 mg/mL protein concentration in 50 mM sodium phosphate pH 7.4. Each experiment was performed adding ANS to a final concentration of 100 µM. After every addition, ANS fluorescence emission spectra were recorded in the 420–550 nm range with excitation at 390 nm, excitation and emission slits were set at 5 nm, with a scanning speed of 50 nm/min. When ANS concentration was 100 µM, its signal was monitored at  490 nm along a temperature ramp starting from 20 to 80 °C (temperature slope 60 °C/hour) in a 1 cm path length cuvette.

### Limited proteolysis

LCs at a concentration of 0.8 mg/mL, were incubated at 37 °C in 50 mM sodium phosphate, 1 M urea at pH 7.4, using a bovine trypsin/LC molar ratio of 1:100. The first sample was collected immediately after trypsin addition and then after 10′, 20′, 30′, 60′, 90′, 120′, 150′, 180′. Subsequently, they were diluted in denaturating and reducing sample buffer (NuPAGE, Invitrogen), heated at 95 °C for 3 min and analysed by SDS-PAGE. Protein bands corresponding to uncleaved LC monomers were quantified by densitometric analysis using Chemidoc^TM^ MP System (Bio-Rad). In order to rule out that the very fast proteolysis of H3, H6, H7, H10 was due to partial or total unfolding several controls were performed in presence of 1 M urea: first the Far-UV spectra are superposable to the ones without urea; secondly the temperature unfolding curves monitored by Far-UV indicate that the unfolding process is starting at a temperature beyond 37 °C. For three LCs (H3, H6 and H9), which displayed very fast kinetics of proteolysis, parallel experiments were performed also in the presence of 0.5 M and 0 M urea, confirming under both conditions a trend for very fast proteolysis for these H LCs (see Figure S1). The latter observations further confirm that the fast kinetics of proteolysis were not artifacts related to excessive urea concentration.

### Crystallization and X-ray structure determination

LCs were crystallized using sitting drops or hanging drops techniques. Each protein was solubilized in 50 mM sodium phosphate pH 7.4 at a concentration of 8.5–10 mg/mL at 20 °C. Crystals were obtained in: H3: 0.1 M Sodium cacodylate pH 6.5, 27% w/v PEG 2000 MME (Stura screen, Molecular dimensions); H6: 0.1 M Bicine pH 9.0, 2% w/v 1,4-Dioxane, 10% w/v PEG 20 K (Crystal Screen I/II, Hampton); H7: 0.1 M HEPES pH7.5, 10% 2-propanol, 20% w/v PEG 4 K (JBS screen, Jena Bioscience); H9: 0.1 M Sodium citrate pH 5.5, 16% w/v PEG 4000, 10% v/v 2-propanol (Stura screen, Molecular dimensions); H10: 0.05 M KBr, 30% PEG 2000 (JCSG screen, Molecular dimensions); M7: 0.1 M MMT (Malic acid, MES and Tris-base buffer) pH 4.0, 25% w/v PEG 1.5 K (microseeding with M8 crystals) (PACT screen, Molecular dimensions); M8: 0.2 M sodium acetate pH 4.6, 2.0 M NaCl (Crystal Screen I/II, Hampton).

Crystals were cryoprotected adding 33% glycerol to mother liquor and then flash frozen in liquid nitrogen. X-ray diffraction data were collected at ESRF (European Synchrotron Radiation Facility of Grenoble–France) at the beam lines: ID29, ID30, ID23-2, BM14. The diffraction data were analysed and processed using MOSFLM and XDS^41,42^, the crystal symmetry was then verified by POINTLESS^43^ and the intensities were merged and scaled with SCALA^44^. The crystal structures were determined by molecular replacement using PHASER, BALBES and MOLREP^45–47^. In order to perform the molecular replacement for H7, the full length LC (pdb: 1JVK)^10^ was used as search model. Then for the subsequent molecular replacements, LC structures determined in house were used. H9 initial model was generated by ARP-wARP^48^. The initial models were subjected firstly to a rigid-body refinement and then to a restrained and TLS refinement using Phenix Refine, Refmac5 and Buster^49–51^. Manual model building, water picking and structure analysis were then performed using Coot^52^. Dimer interface analysis shown in Table S3 was performed using PISA^53^. Fab elbow angles were calculated with phenix.fab_elbow_angle^49^. Figures of crystallographic structures were done using PyMOL and CCP4mg^54^.

The atomic coordinates and the structure factors of the seven structures of LCs have been deposited in the Protein Data Bank with the following accession numbers: 5MTL (H3), 5MUD (H6), 5MUH (H7), 5M6A (H9), 5M76 (H10), 5MVG (M7), 5M6I (M8).

## Electronic supplementary material

Supplementary information
