# Supplementary material for: Concurrent structural and biophysical traits link with immunoglobulin light chains amyloid propensity
Source: Sci Rep. 2017 Dec 1;7:16809. doi: 10.1038/s41598-017-16953-7 (PMC5711917; doi:10.1038/s41598-017-16953-7)
Supplement: Supplementary file 1 — Supplementary information [file 41598_2017_16953_MOESM1_ESM.doc]

**Concurrent structural and biophysical traits link with immunoglobulin light chains amyloid propensity**

Luca Oberti^1^, Paola Rognoni^2^, Alberto Barbiroli^3^, Francesca Lavatelli^2^, Rosaria Russo^1^, Martina Maritan^1^, Giovanni Palladini^2^, Martino Bolognesi^1^, Giampaolo Merlini^2^ and Stefano Ricagno^1^*

Supplementary information

|  | | **Clinical features** | | | | | | | | **Cardiac features** | | | | | |
| --- | --- | --- | --- | --- | --- | --- | --- | --- | --- | --- | --- | --- | --- | --- | --- |
| **LC**  **code** | **Germline** | **Gender,**  **age** | **Diagnosis** | **Organs involved** | **Serum λ FLC**  **(mg/l)** | **κ/λ FLC ratio** | **dFLC (mg/l)** | **Proteinuria**  **(g/24h)** | **Creatinine**  **(mg/dl)** | **Cardiac stage°** | **NT-proBNP (ng/l)BNP* (ng/L)** | **cTnI**  **(ng/ml)** | **IVS**  **(mm)** | **PW**  **(mm)** | **EF**  **(%)** |
| **H3** | 1c  (IGLV1-44) | M, 65 | AL | H | 252 | 0.081 | 231.5 | 0.18 | 1.76 | III | 4491 | 0.35 | 16 | 16 | 42 |
| **H6** | 1b  (IGLV1-51) | F, 72 | AL | H, K, PNS | 248 | 0.05 | 235 | 0.7 | 0.81 | III | 18731 | 0.207 | 18.5 | 16 | 50 |
| **H7** | 1b  (IGLV1-51) | M, 45 | AL | H | 477 | 0.01 | 469 | 0.33 | 0.98 | III | 8882 | 0.16 | 19 | 19 | 45 |
| **H9** | 2c  (IGLV2-8) | M, 59 | AL | H, ST | 699 | 0.02 | 689 | 8 | 1.19 | III | 3530 | 0.152 | 17.5 | 17 | 50 |
| **H10** | 1a  (IGLV1-36) | M, 73 | AL | H, L | 475 | 0.04 | 454.5 | 0.16 | 1.07 | III | 7296 | 0.347 | 15.5 | 15 | 45 |
| **H15** | 6a  (IGLV6-57) | M, 53 | AL | H, PNS, ST | 839 | 0.002 | 837.5 | 0.98 | 0.74 | III | 1444 | 0.222 | 16.8 | 16 | 70 |
| **H16** | 2a2  (IGLV2-14) | M, 72 | AL | H, K | 383 | 0.05 | 363.6 | 8.1 | 2.73 | III | 1926* | 1.188 | 14.9 | 14.4 | 61 |
| **H18** | 3l  (IGLV3-19) | M, 69 | AL | H, ST, PNS | 509 | 0.01 | 500.4 | 0.82 | 0.97 | III | 3839 | 0.345 | 21.5 | 18 | 61 |
| **M2** | 2b2  (IGLV2-23) | M, 65 | MM  (follow-up: 1.8 y) | - | 1140 | 0.001 | 1138.5 | 0.12 § | 0.89 | n.a. | 201 | n.a. | 11 | 11 | 65 |
| **M7** | 3l  (IGLV3-19) | F, 71 | MM  (follow-up: 5.2 y) | - | 6130 | 0.001 | 6124 | 0.52 § | 2.07 | n.a. | 42* | 0.007 | 9 | 9 | 65 |
| **M8** | 2b2  (IGLV2-23) | M, 48 | MM  (follow-up: 5.6 y) | - | 573 | 0.011 | 567 | 1.87§ | 0.84 | n.a. | 14.5 | 0.003 | 10 | 10.5 | 67 |
| **M9** | 2b2  (IGLV2-23) | M, 61 | MM  (follow-up: 2.7 y) | - | 8510 | 0.00 | 8508 | 7.24 § | 4.92 | n.a. | 74* | 0.021 | 10.5 | 10.5 | 62 |
| **M10** | 2a2  (IGLV2-14) | M, 55 | MM  (follow-up: 1.6 y) | - | 12200 | 0.00 | 12198.5 | 0.9 § | 1.1 | n.a. | 88.2 | 0.014 | 11 | 10.5 | 65 |

**Table S1 (Previous page):** Main clinical and biochemical characteristics of the light chains used in this study, at the time of diagnosis and urine collection. Multiple myeloma patients did not show evidence of amyloid deposits or amyloid organ involvement throughout the follow-up time (indicated in years). § Entirely constituted by Bence-Jones proteinuria (monoclonal urinary free light chains). °According to Gertz *et al*. [^1^](#_ENREF_1). Abbreviations: M, male; F, female; H, Heart; K, Kidney; ST, Soft Tissues; PNS, Peripheral Nervous System; BJ, Bence Jones; FLC, Free Light Chains; BNP, Brain Natriuretic Peptide; cTnI, cardiac Troponin I; IVS, Interventricular Septum; PW, Posterior Wall; EF, Ejection Fraction; n.a., not applicable.

Reference ranges: serum λ FLC <26.3 mg/l, κ/λ ratio 0.26-1.65; serum creatinine <1.18 mg/dl in men, <1.02 mg/dl in women; NT-proBNP 5 <332 ng/l; BNP, <99 ng/l; cTnI <0.04 ng/ml.

**Table S2 (next page):** Data collection and refinement statistics.

a Rmerge = Σhkl Σj|Ihkl,j - <Ihkl>|/ΣhklΣjIhkl,j , where Ihkl is the observed intensity and <Ihkl> is the average intensity for the hkl reflection.

b Rwork = Σhkl|Fo - Fc|/ΣhklFo for all data except 5–10%, which were used for the R_free_ calculation.

Values given in parenthesis refer to the high-resolution shell.

| **Structure** | **H3** | **H6** | **H7** | **H9** | **H10** | **M8** | **M7** |
| --- | --- | --- | --- | --- | --- | --- | --- |
| **Beam Line** | ID23-2 (ESRF) | ID30 (ESRF) | ID29 (ESRF) | ID29 (ESRF) | ID29 (ESRF) | ID 23-1 (ESRF) | ID29 (ESRF) |
| **Space group** | P1 | I 1 2 1 | C2 | P 1 | P 21 21 21 | I 4 2 2 | P 31 2 1 |
| **Unit cell constants (Å)** | a = 46.48, b = 65.08  c =88.39,  α= 84.96° β = 86.66° γ=79.93° | a = 80.06 , b = 72.81  c = 84.99,  α= 90° β = 105.29°  γ= 90° | a = 100.01, b = 72.82  c = 73.72,  α= 90° β = 121.19°  γ= 90° | a = 46.49 , b = 63.43  c = 86.64,  α= 95.32° β = 85.84°  γ= 99.58° | a = 55.49 , b = 71.42  c = 103.93,  α= 90° β = 90° γ= 90° | a = 229.38 , b = 229.38  c = 64.43,  α= 90° β =90 ° γ= 90° | a = 70.72 , b = 70.72  c = 171.95,  α= 90° β =90 ° γ= 120° |
| **Resolution (Å)** | 28.57 – 2.45  (2.54 – 2.45) | 52.98 – 2.34  (2.42 – 2.34) | 55.45 – 2.70  (2.74 – 2.70) | 45.77 – 1.64  (1.70 – 1.64) | 48.95 - 2.50 (2.60 - 2.50) | 57.3 – 2.20  (2.32 – 2.20) | 57.70 – 2.20  (2.27 – 2.20) |
| **R_merge_ (%)** | 12.6 (61.0) | 4.5 (51.2) | 7.7 (57.4) | 8.7 (54.0) | 10.8 (51.0) | 27.1 (254.8) | 7.70 (53.6) |
| **I/σI** | 8.8 (2.2) | 16.6 (2.5) | 7.9 (1.6) | 7.3 (1.6) | 8.6 (2.3) | 12.4 (2.3) | 26.0 (4.6) |
| **Completeness (%)** | 98.2 (97.9) | 96.0 (95.8) | 97.0 (98.2) | 95.7 (94.2) | 99.2 (97.9) | 99.7 (99.6) | 100.0 (100.0) |
| **Multiplicity** | 3.3 (3.2) | 3.5 (3.5) | 3.5 (3.2) | 3.6 (3.7) | 4.3 (4.1) | 14.2 (14.6) | 10.9 (11.1) |
| **Unique reflections** | 36581 (3606) | 19281 (1908) | 12871 (1137) | 113746 (11177) | 14723 (1407) | 43679 (6279) | 26134 (2224) |
| **Refinement** |  |  |  |  |  |  |  |
| **R_work_ (%)** | 23.42 | 20.70 | 26.92 | 18.01 | 23.87 | 19.50 | 22.15 |
| **R_free_ (%)** | 27.68 | 25.00 | 32.10 | 20.07 | 28.24 | 22.65 | 25.14 |
| **Average B factor, all atoms (Å^2^)** | 38.5 | 59.7 | 48.5 | 25.7 | 38.2 | 30.6 | 47.7 |
| **Number of atoms** | 5940 | 3143 | 2718 | 7156 | 3169 | 3419 | 3134 |
| **Protein** | 5896 | 3121 | 2692 | 6235 | 3110 | 3158 | 2870 |
| **Waters** | 44 | 22 | 26 | 882 | 49 | 259 | 258 |
| **Heteroatoms** | - | - | - | 39 | 1 | 2 | 6 |
| **Ramachandran plot, *n* (%)** |  |  |  |  |  |  |  |
| **Most favoured region** | 94.97 | 95.95 | 91.0 | 97.51 | 97.13 | 97.86 | 94.92 |
| **Allowed region** | 4.51 | 3.57 | 8.0 | 2.49 | 2.87 | 2.14 | 5.08 |
| **Outliers** | 0.52 | 0.48 | 1.0 | 0 | 0 | 0 | 0 |
| **Elbow Angle (°)** | 116.7 | 108.0 | 124.0 | 116.2 | 112.7 | 157.4 | 127.7 |

| **RMSD**  (Å/Cα) | **H3** | **H6** | **H7** | **H9** | **H10** | **M8** | **M7** |
| --- | --- | --- | --- | --- | --- | --- | --- |
| **H3** | ——— |  |  |  |  |  |  |
| **H6** | 0.50/196 | ——— |  |  |  |  |  |
| **H7** | 0.87/188 | 0.61/188 | ——— |  |  |  |  |
| **H9** | 0.56/196 | 0.58/198 | 1.05/188 | ——— |  |  |  |
| **H10** | 0.70/194 | 0.63/196 | 1.60/189 | 0.61/196 | ——— |  |  |
| **M8** | 0.53/196 | 0.56/198 | 0.61/189 | 0.50/198 | 0.59/196 | ——— |  |
| **M7** | 0.72/192 | 0.62/194 | 0.78/189 | 0.62/194 | 0.48/194 | 0.56/194 | ——— |

**Table S3A:** Root mean square Cα deviations (r.m.s.d.) calculated for the superposition of C_l_ dimers.

| **RMSD**  (Å/Cα) | **H3** | **H6** | **H7** | **H9** | **H10** | **M7** | **M8** |
| --- | --- | --- | --- | --- | --- | --- | --- |
| **H3** | ——— |  |  |  |  |  |  |
| **H6** | 0.79/92 | ——— |  |  |  |  |  |
| **H7** | 0.67/87 | 0.87/92 | ——— |  |  |  |  |
| **H9** | 0.78/93 | 0.77/107 | 0.81/92 | ——— |  |  |  |
| **H10** | 0.83/92 | 0.75/108 | 0.90/92 | 0.75/105 | ——— |  |  |
| **M7** | 1.23/70 | 0.97/105 | 0.107/91 | 0.83/103 | 0.98/104 | ——— |  |
| **M8** | 0.74/93 | 0.84/105 | 0.77/92 | 0.62/107 | 0.80/103 | 0.90/104 | —--- |

**Table S3B:** R. m. s. d. calculated for the superposition of V_l_ monomers.

| **RMSD**  (Å/Cα) | **H3** | **H6** | **H7** | **H9** | **H10** | **M8** |
| --- | --- | --- | --- | --- | --- | --- |
| **H3** | ——— |  |  |  |  |  |
| **H6** | 3.06/171 | ——— |  |  |  |  |
| **H7** | 1.06/168 | 2.95/161 | ——— |  |  |  |
| **H9** | 1.28/186 | 2.58/174 | 1.10/171 | ——— |  |  |
| **H10** | 1.41/188 | 3.55/183 | 1.44/172 | 1.68/210 | ——— |  |
| **M8** | 3.06/140 | 2.65/129 | 3.46/122 | 2.80/151 | 2.72/152 | ——— |

**Table S3C:** R.m.s.d. calculated for the superposition of V_l_ dimers. The M7 structure has not been used in this calculation given the partial traceability of one of the V_l_ domains.

| **LC** | **Solvation energy, chain A* (kcal/mol)** | **Solvation energy, chain B* (kcal/mol)** | **Dimer interface chain A (Å^2^)** | **Dimer interface chain B (Å^2^)** |
| --- | --- | --- | --- | --- |
| **H3** | -9.2 | -9.3 | 1504 | 1486 |
| **H6** | -7.4 | -8.2 | 1564 | 1568 |
| **H7** | -8.7 | -7.3 | 1444 | 1347 |
| **H9** | -8.6 | 10.8 | 1577 | 1569 |
| **H10** | -12.1 | -11.3 | 1670 | 1671 |
| **M7** | -10.2 | -9.1 | 1336 | 1568 |
| **M8** | -6.8 | -8.7 | 1137 | 1125 |

**Table S4:** dimer interface analysis. (*): Energy gain on complex formation.


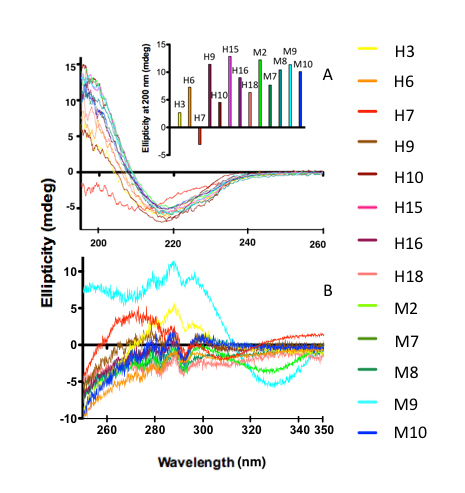


**Figure S1:** Inspection of LC secondary and tertiary structures. (A) panel A shows the Far-UV CD spectra: all LCs display spectra with quite comparable shapes, typical of proteins with high β-structure content: unexpectedly, the H7 spectrum, which however is well reproducible, is atypical and not superposable to all other spectra. Since H7 was successfully crystallized (see below) and showed cooperative unfolding curves (Figure 2), we can exclude that this spectrum indicates an aggregated or an unfolded species. Closer inspection of all the LC spectra shows that M LCs cluster together, displaying, on average, a slightly stronger CD signal (inset), as if the M LCs were typically characterized by an overall slightly more regular β-structure in solution. (B) Near-UV CD spectra are considered a tertiary structure fingerprint. The well conserved shape shared by the recorded protein spectra, suggests that LCs maintain a comparable structure in solution. However, both the number of near-UV chromophores (mainly Trp and Tyr residues) and their chemical environments are not strongly conserved in different LCs due to sequence variability in the V_L_ domains. Therefore, the resulting near-UV CD spectra show expected, although unpredictable, LC-specific differences, *e.g.* the M9 spectrum is more intense than all the other LCs.


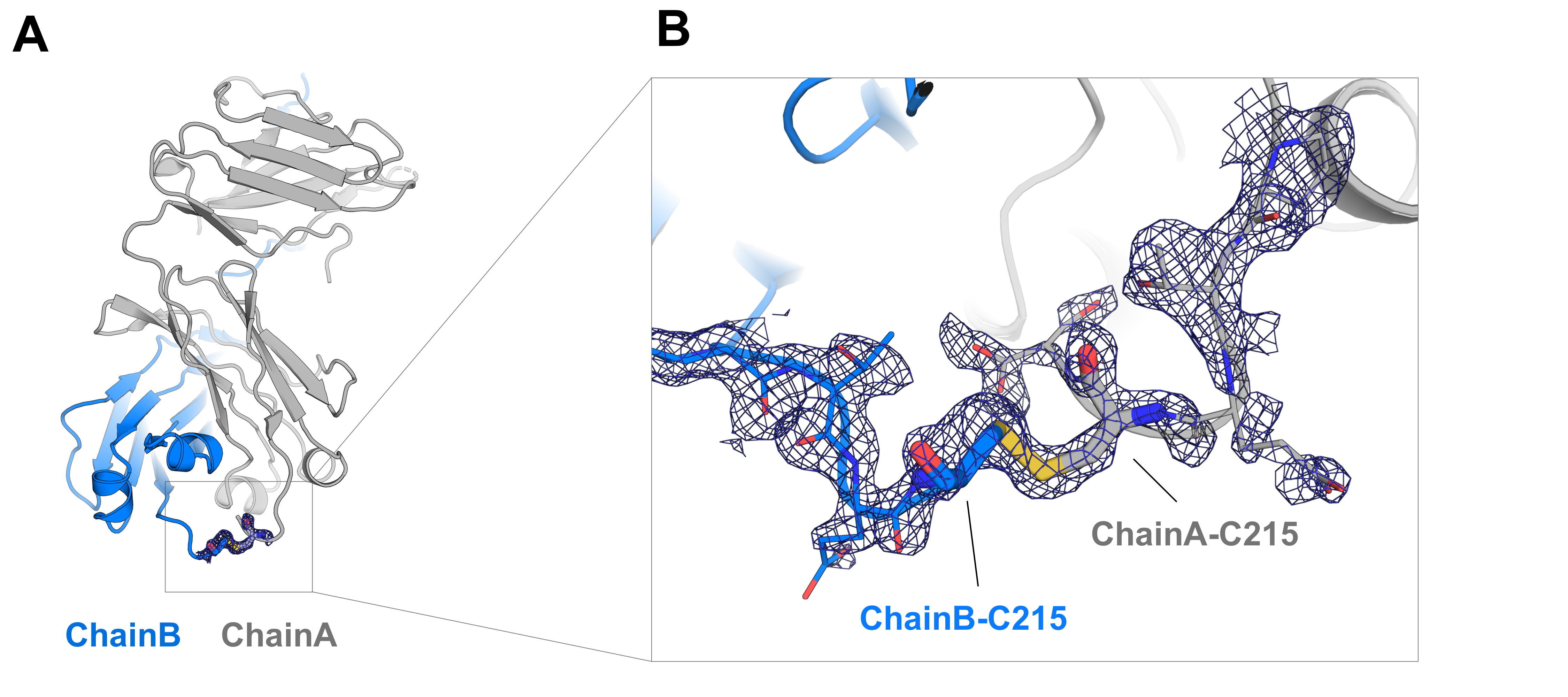


**Figure S2**: Intermolecular disulphide bond in the H9 dimer. (A) Cartoon representation of the overall fold of the H9 homodimer, having at the C-terminal a disulphide bond linking C215 the two LC monomers (ChainA and ChainB, colored in grey and blue, respectively). (B) Zoom into the well-defined electron density of C215-C215 disulphide bond (sticks representation) and the surrounding C-terminal residues (lines representation). 2*F*o-*F*c electron density map is contoured at 1σ.


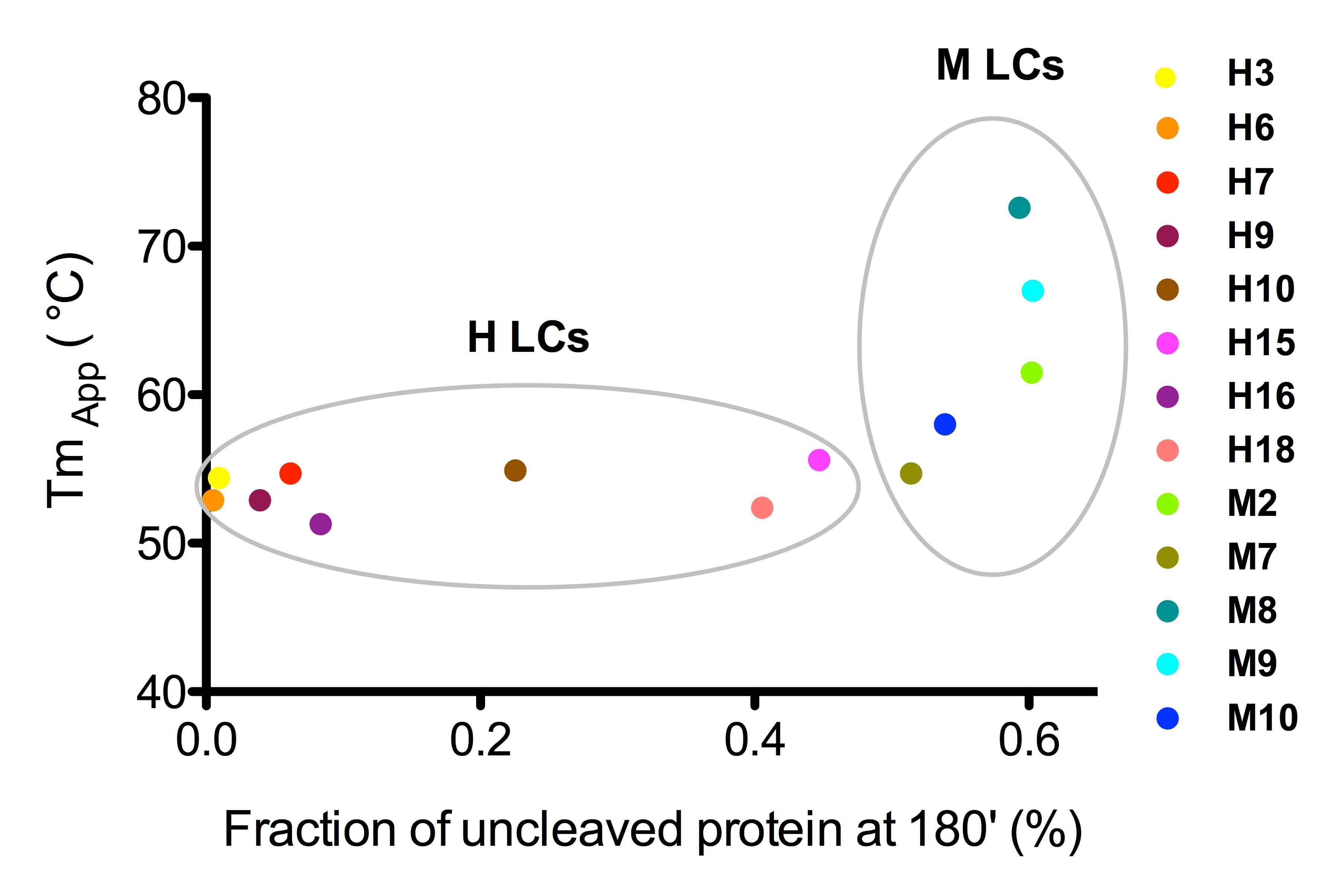


**Figure S3**: Plot of uncleaved LC at the end of the proteolysis experiments as shown in Figure 3A (180 minutes) against apparent melting temperatures (Tm_app_) monitored by Far-UV (see Table 2). H and M-LCs are colour coded as in Figures 2 and 3. The H and M LC sets are circled highlighting the clustering between H and M LCs.


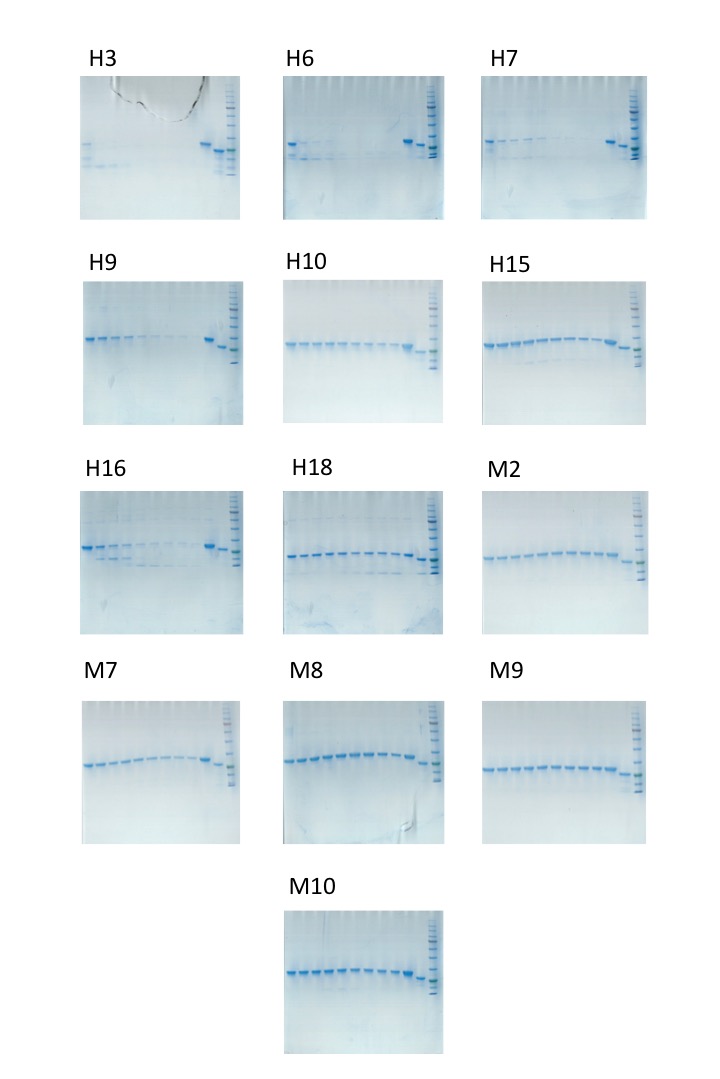


**Figure S4**: Raw images of the SDS-PAGE shown in Figure 3

**References:**

1 Gertz, M. A. & Merlini, G. Definition of organ involvement and response to treatment in AL amyloidosis: an updated consensus opinio*n Amyloid : the international journal of experimental and clinical investigation : the official journal of the International Society of Amyloidos*i**s** 17, 48 (2010).
